# Return-to-Work After Carpal Tunnel Release Across Surgical Techniques: A Narrative Review

**DOI:** 10.3390/medicina62030557

**Published:** 2026-03-17

**Authors:** Christian A. Lobos, Kyle Wilcox, Thomaz De Campos Silva, Shea Wilcox

**Affiliations:** 1 Inner North Medical Clinic, Melbourne, VIC 3057, Australia; 2 Melbourne Specialty Centre, Melbourne, VIC 3056, Australia; 3 Carpal Tunnel Institute, Melbourne, VIC 3056, Australia; 4 Carpal Tunnel Australia, Adelaide, SA 5000, Australia; 5 Carpal Tunnel Australia, Brisbane, QLD 4000, Australia; 6 Carpal Tunnel Australia, Canberra, ACT 2600, Australia; 7 Kangaroo Point Medical Centre, Brisbane, QLD 4169, Australia

**Keywords:** carpal tunnel syndrome, Return-to-Work, carpal tunnel release, ultrasound-guided surgery, minimally invasive surgery, microinvasive surgery

## Abstract

Carpal Tunnel Syndrome (CTS) is the most common upper-limb entrapment neuropathy and remains a major contributor of work-related disability. While Carpal Tunnel Release (CTR) reliably improves symptoms, functional recovery is less consistently reported. Return-to-Work (RTW) has emerged as a practical functional outcome, but definitions and reporting remain heterogeneous. We conducted a narrative review of English-language studies reporting RTW or work-absence outcomes following open, endoscopic, ultrasound-guided, ultraminimally invasive, and microinvasive CTR techniques. Due to variability in study design and RTW definitions, findings were synthesised descriptively. Across the literature, RTW durations progressively shortened as procedural invasiveness decreased. Open CTR commonly reported RTW at four to six weeks, endoscopic techniques at two to four weeks, and ultrasound-guided approaches at approximately 10–21 days. Recent ultraminimally invasive and microinvasive systems frequently reported RTW within one to two weeks when performed under local anaesthesia. These findings highlighted RTW as a meaningful functional benchmark and underscore the role of imaging-guided access minimisation in accelerating postoperative recovery.

## 1. Introduction

Carpal Tunnel Syndrome (CTS) is the most common entrapment neuropathy of the upper limb, affecting 3% to 5% of adults in the general population [1] and remaining one of the most frequent work-related symptoms worldwide [2]. Incidence varies by occupation and age, especially where repetitive strain is common, and several population-based studies show that the demand for surgical treatment continues to rise [2,3,4]. CTS contributes to musculoskeletal morbidity and is linked with lost productivity and long-term compensation expenditure [5,6]. As such, CTS represents a substantial musculoskeletal burden for functional capacity and work participation.

Carpal Tunnel Release (CTR) remains the preferred intervention for moderate to severe or refractory CTS, with long-term studies demonstrating durable symptom relief and minimal recurrence rates [7]. Over time, the criteria used to judge postoperative “success” have changed [8,9,10]. Traditional outcomes such as symptom scores, grip strength or nerve-conduction studies remain useful, but they often fail to show how well patients resume work or usual activities. This gap has led many groups to incorporate Return-to-Work (RTW) duration as a complementary functional measure, reflecting recovery in a way that aligns more closely with daily life and socioeconomic impact [11,12].

Recent cohort studies and registry studies showed that RTW is influenced by more than the surgical technique. Factors such as pain, hand function, workplace demands, job flexibility and compensation status all influenced when patients actually return to work [5,11,12]. For example, people in manual roles often returned later than those in desk-based positions. As a result, RTW provides insight into both clinical recovery and the broader practical consequences of CTR. Delayed RTW contributed to indirect costs, prolonged healthcare use and downstream occupational disruption [5,6].

Comparative studies across CTR modalities consistently showed shorter RTW intervals with less invasive approaches [13,14,15,16,17,18,19,20], although the size of differences varied between cohorts. Historically, open CTR (OCTR) involved larger incisions and required approximately four to six weeks before full-duty RTW [13]. Endoscopic techniques brought this down to about two to four weeks. Much of this improvement likely relates to reduced dissection and fewer wound-related symptoms [14]. Ultrasound-guided CTR (US-CTR) further shortened the interval, with median RTW durations of 10–21 days and comparable safety profiles [15,16,17,18]. These findings later informed the development of ultraminimally invasive and microinvasive techniques, which continue to prioritise decompression through increasingly small access routes [21,22,23].

Recent work has focussed on methods that achieve complete ligament release through small punctures under real-time imaging guidance. Needle-mounted blade systems developed through cadaveric and clinical testing allowed full decompression through a single-entry point, typically under local anaesthesia. Published series reported RTW of approximately seven days, with outcomes similar to open or endoscopic CTR (ECTR) in terms of symptom relief and durability [19,20,24,25]. Thread-based and hook-knife systems are emerging related approaches. Their goal is similar as each aims to limit access trauma while still achieving full TCL release, but they differ in mechanics and learning curves [26,27].

This review examined the development of CTR with RTW as the central comparative outcome. Beyond the anatomical and technical changes, we highlighted how each method influences postoperative recovery in practical terms: how soon people get back to work, how their symptoms change, and which factors seem to influence recovery. In particular, this review highlighted how advances in imaging, especially real-time ultrasound, have enabled progressive reductions in surgical access while preserving anatomical safety and procedural control. By tracing CTR through an imaging-informed lens, RTW is used as a practical marker of how visualisation strategy influences recovery timelines.

The purpose of this review is to synthesis the evolution of CTR techniques using RTW duration as a central functional benchmark, and to examine how access minimisation and imaging-guided visualisation have influenced recovery timelines

## 2. Materials and Methods

### Literature Identification and Narrative Synthesis Approach

This review was conducted as a narrative synthesis of the literature examining CTR techniques and their associated RTW outcomes. PubMed, Embase, Scopus, Google Scholar, and JSTOR were searched for English-language studies describing open, endoscopic, ultrasound-guided, ultraminimally invasive, and microinvasive CTR techniques. Particular attention was given to reports including RTW or work-absence outcomes.

Historical and foundational studies were included to contextualise the evolution of CTR techniques. Given the heterogeneity in RTW definitions, study designs, occupational stratification, and follow-up intervals, formal meta-analysis was not undertaken. Instead, studies were compared descriptively, with RTW used as a functional benchmark across techniques. Emphasis was placed on imaging guided approaches, particularly ultrasound-guided techniques, to evaluate how visualisation strategy and access minimisation have influenced procedural precision and functional recovery.

For the purposes of this review, RTW was interpreted as the reported duration from surgery to resumption of occupational activity, whether defined as light-duty or full-duty return; recognising definitions, reporting varied between studies.

## 3. Epidemiology and Socioeconomic Burden

CTS remains a significant public health and occupational issue due to its prevalence and the wide range of work environments in which it appears. Estimates suggest a prevalence of approximately 3.8% of women and 2.7% of men, with rates increasing with age and repetitive strain exposure [1]. Several studies consistently identified that repetitive, hand intensive work and sustained computer use are frequently implicated, while exposure to vibrating tools added additional risk in some industries [2]. As a result, CTS is widely recognised as the most common upper-limb entrapment neuropathy and a major contributor to work-related musculoskeletal complaints [28].

Longitudinal reports from various health systems also showed that the number of patients seeking surgical decompression continues to rise [4,7]. In a 10.5-year New Zealand review of 2309 patients, English and Gwynne-Jones reported an incidence rate of 14.8 per 10,000 person-years, noting increases in bilateral and recurrent cases [4]. Such findings highlight the burden placed on both primary care and surgical services.

From an occupational standpoint, CTS has long been associated with cumulative trauma and repetitive wrist-loading. Early clinical reviews classified CTS as an occupational disorder in settings involving forceful gripping or repetitive wrist flexion–extension cycles [2]. Subsequent Australian data also linked hand–arm vibration exposure with an increased incidence of CTS among industrial workers. More recent analyses have expanded this view, noting that service workers and office-based employees (particularly those with prolonged computer use) may also be affected [2,28]. The economic implications are considerable: CTS may contribute to lost productivity, prolonged work absence and substantial compensation costs [5,29].

Economic evaluations reinforce this burden. Delays in RTW after CTR can generate sizeable earnings losses and compensation expenditure [6,30]. A Washington state study estimated lifetime earnings losses exceeding US$40,000 for CTS claimants who remained symptomatic or did not undergo timely surgery [31]. In the United Sates, total societal costs attributed to CTR approach US$2 billion annually, with indirect costs such as lost wages representing a major component [32]. Cost-effectiveness analyses supported CTR over non-operative management, showing meaningful long-term savings by reducing chronic disability and facilitating earlier RTW [33].

Recent cohort studies also suggested that prolonged RTW after CTR often reflects non-surgical factors rather than operative shortcomings. Socioeconomic variables, illness perception and compensation status show strong associations with delayed RTW [5,34,35]. These findings emphasise that CTS imposes not only direct healthcare costs but also indirect economic losses. Early, effective CTR may mitigate these effects by restoring work capacity and reducing the duration of rehabilitation.

## 4. Historical and Contextual Background of Carpal Tunnel Release

CTR developed over the past century as clinicians looked for practical ways to relieve median nerve compression within the carpal tunnel. Recognition of CTS as a distinct condition emerged in the early 20th century, during a period when peripheral nerve surgery and occupational health were both evolving. By the 1960s, CTR had become a familiar procedure, supported by anatomical descriptions and accumulating clinical experience. Much of this early work still shapes contemporary approaches to decompression.

### 4.1. Early Recognition and Pathophysiological Understanding

The first steps toward CTR were rooted in broader work on peripheral nerve entrapments. In 1933, Sir James Learmonth outlined the concept of operative decompression [36,37]. This idea was later carried across to the wrist when Brain, Wright and Wilkinson published their landmark 1947 report describing six patients with “spontaneous compression of the median nerve” [38]. They identified the TCL as the main point of constriction and showed that dividing it relieved symptoms. This established CTS as a mechanical entrapment neuropathy and set the basis for treating it through direct decompression.

### 4.2. The First Open Carpal Tunnel Releases

CTR became a more reproducible operation through the work of George S. Phalen, who refined diagnostic criteria and published outcomes over nearly two decades. In his reports on more than 600 hands, Phalen described a longitudinal palmar incision with complete TCL division and noted durable symptom relief with reduced rate of recurrence [39,40]. Around the same time, Radford Tanzer provided additional confirmation through combined clinical and anatomical study, reporting consistent sensory and motor improvement after decompression [41]. Surgical safety improved further when Ulrich Lanz documented variants of the median nerve within the carpal tunnel [42], helping surgeons avoid inadvertent nerve injury. Together, these contributions established OCTR as the standard technique for many years.

### 4.3. From Wide Exposure to Minimisation

Although OCTR was widely adopted [9], its relatively large incision and associated soft-tissue disruption raised concerns about postoperative discomfort and time away from work. Several clinical series reported longer convalescence with wider exposures [43,44]. These observations prompted attempts to reduce dissection while still achieving full TCL release. Earlier smaller-incision approaches and the introduction of endoscopic techniques drew on the anatomical knowledge refined by Phalen [39,40] and Lanz [42]. Studies by Agee and colleagues [45] and later by Kerr and co-workers [46] showed that ECTR could provide symptom relief comparable to OCTR while reducing wound-related symptoms and shortening recovery. This added practical support for progressively limiting surgical exposure where feasible.

### 4.4. Enduring Contributions of Early Innovators

The work of these early investigators continues to underpin modern CTR. Brain and colleagues clarified the site of compression [38], Phalen established the operative method and its expected outcomes [39,40], Tanzer demonstrated clinical effectiveness [41], and Lanz’s classification still informs safe surgical exposure [42]. Their contributions are frequently cited in contemporary discussions of CTR. Later historical analyses highlighted that what is considered the “first CTR” reflects this early combination of anatomical insight and practical decompression [37]. As CTR became routine, early follow-up studies established typical timelines for functional recovery, with many patients regaining hand use within four to six weeks [39,40,41]. These intervals became the comparative reference for assessing newer techniques, particularly those aiming to shorten rehabilitation and promote earlier RTW. They also marked a shift from evaluating CTR solely on symptom improvements to considering how quickly patients could return to everyday function.

## 5. Early Return-to-Work Evidence

Early studies on recovery after CTR mainly concentrated on symptoms, nerve-conduction changes and complications. Even so, several reports from the 1970s through the 1990s made incidental observations about when patients returned to work, although RTW was not the focus of these publications. Much of this work suggested that hand pain, wound sensitivity and reduced grip strength were the main issues delaying a patient’s ability to resume their job. Phalen’s clinical reports provided some of the first indications of symptom relief related to functional recovery. He did not measure RTW directly, but many patients were described as returning to activity within a few weeks once wound tenderness had settled [39,40]. Tanzer reported a similar pattern: improvement in median nerve function and nocturnal symptoms often coincided with a gradual return to manual work [41]. These observations reflected priorities of the time, where symptom improvement was the primary endpoint and work integration was noted only in passing.

More structured RTW data started appearing in later publications. One of the earliest prospective comparisons, by Cook and colleagues [43], examined outcomes after different approaches to OCTR and found that larger incisions and prolonged palmar tenderness were associated with longer delays before returning to work. Chaise et al. [44] reported comparable findings, noting that wider exposures were often followed by a slower return to full-duty roles. These studies highlighted that the amount of surgical dissection, not just symptom severity, could influence how long patients remained out of work. They also helped explain why RTW began to appear as a relevant measure of postoperative recovery, particularly for patients in physically demanding occupations.

With the introduction of less invasive techniques, early comparative studies often included RTW as a secondary measure. Several reports on ECTR documented reduced postoperative discomfort and earlier functional use of the hand, which in practice translated to shorter RTW intervals than those typically described in open procedures [45,46]. Definitions of RTW varied widely, but a general trend favouring reduced exposure was already noticeable. These early comparisons introduced the idea that technical modifications aimed at minimising tissue trauma could have meaningful effects on everyday function. Taken together, the early literature repeatedly showed that wound discomfort and grip weakness were major barriers to returning to work, that larger incisions tended to delay recovery, and that the shift towards less invasive techniques corresponded with shorter RTW intervals. Although many studies were limited by inconsistent RTW definitions and occupational stratification, they provided the first practical benchmarks against which modern minimally invasive, ultrasound-guided and microinvasive CTR techniques are now assessed.

## 6. Minimally Invasive and Endoscopic Techniques

By the 1990s it had become clear that, for many patients, the main limitation after OCTR was not the ligament release but the incision and its after-effects. Pillar pain, scar tenderness, and slower grip recovery were frequently mentioned in follow-up studies [47,48]. These observations led surgeons to question how much exposure was actually needed to divide the TCL safely, and this gradually pushed the field toward smaller incisions and eventually endoscopic access.

### 6.1. The Rise in Endoscopic Approaches

ECTR altered expectations around the early phase of recovery. Chow’s early work [49,50,51], along with reports from Okutsu [52] and later Agee et al. [45], demonstrated that the TCL could be divided through small portals while still maintaining internal visualisation. The motivation was straightforward: if the surgeon could see the ligament internally, a large palmar incision seemed unnecessary. Clinical studies reflected this shift. Randomised and multicentre trials showed that patients who underwent endoscopic release often regained hand comfort and grip strength sooner than those treated with open techniques, and RTW trends followed a similar pattern [45,46,53,54]. Typical RTW durations fell around two to four weeks, compared with the four to six weeks commonly documented after OCTR. For patients in physically demanding occupations, this difference had immediate practical relevance.

### 6.2. Complications and the Learning Curve

ECTR did not gain immediate universal acceptance. Early reports raised concerns about nerve irritation, incomplete release and the steep learning curve associated with the new equipment [47,48]. Centres adopting the technique for the first time encountered more transient complications, and this understandably tempered enthusiasm. Over time, however, follow-up studies showed that once surgeons gained adequate experience, complication rates were comparable to OCTR [55]. Improvement in optics, instrumentation and blade housings also reduced the difficulty [56,57]. By the mid-2000s, the balance of evidence and practical experience had made ECTR a routine option in many settings.

### 6.3. Return-to-Work as a Functional Outcome

As comparative studies accumulated, RTW gained a more explicit role in evaluating CTR outcomes. Sanati et al. [6] reported that endoscopic release shortened RTW by roughly one-third compared with open surgery. Other studies pointed out that occupation, workplace structure and compensation factors could still influence the actual timing of return [3,35], but the general pattern held: patients treated with less invasive methods often resumed work sooner.

### 6.4. The Transitional Step Toward Ultraminimal Access

Mini-open approaches, including the Knifelight^®^ and other sub-2 cm incision techniques, provided an intermediate option between open and fully endoscopic procedures. These approaches maintained direct visualisation while reducing palmar dissection, and several studies documented RTW durations approaching those seen after ECTR [13,56,57,58]. Although they did not replace ECTR in most institutions, these techniques helped reinforce a principle that shaped subsequent innovations: reducing surgical exposure, as long as the ligament is fully released, tends to support faster recovery. This reasoning directly informed the development of ultrasound-guided, thread-based and microinvasive systems that followed.

## 7. Ultrasound-Guided Minimally Invasive Carpal Tunnel Release

The integration of high-resolution ultrasound into CTR changed how many surgeons approached the procedure. Unlike optical or landmark-based techniques, ultrasound provides dynamic visualisation of the transverse carpal ligament, median nerve, and adjacent structures throughout each procedural step, fundamentally altering the relationship between access size, safety, and recovery. Instead of relying on surface landmarks or working through a viewing portal, US-CTR made it possible to visualise the TCL, median nerve and relevant anatomical variants in real time throughout the release. Teams adopting this approach reported that being able to follow each step of the procedure, not just the point of ligament division, improved anatomical orientation and control compared with earlier methods [18,22,59].

### 7.1. Visual Control and Anatomical Safety

One of the early concerns with limited-exposure CTR was the risk of injury to the median nerve or its branches. Ultrasound addressed this issue directly. Continuous dynamic imaging allows identification of the median nerve, bifid variants, persistent median arteries and the superficial palmar arch before any cutting is performed [22,60]. Beyond procedural guidance, contemporary ultrasound assessment of CTS extends beyond anatomic localisation alone. Recent evidence demonstrated that multiparametric ultrasound, including morphometric measures, such as cross-sectional area and nerve rations, dynamic evaluation of median nerve motion, microvascular imaging, and elastographic assessment of nerve stiffness, can provide complimentary information on nerve pathology, severity, and biomechanical impairment [61]. These developments position ultrasound not only as a guidance tool for decompression but also as a modality capable of characterising disease severity and structural compromise prior to intervention. This capability altered how the procedure was performed. Rather than advancing toward the ligament based on indirect cues, the operator could observe the relationship between the instrument and the TCL throughout the manoeuvre. Studies of US-CTR have reported few complication rates and consistent completeness of TCL division [62]. Further investigations confirmed full decompression with minimal soft-tissue disruption, supporting the anatomical reliability of the approach [63].

### 7.2. Functional Recovery and Return-to-Work Outcomes

Functional recovery after US-CTR follows a consistent pattern across published cohorts. Reported RTW durations fall between 10 and 21 days, shorter than those described in many endoscopic and mini-open series [22,64,65]. These results coincide with real-world registry data encompassing 1256 consecutive US-CTR procedures, where >90% of patients resumed work inside three weeks [66]. Patients typically reported less postoperative pain and resumed light activity within days, likely related to the smaller incision and avoidance of palmar dissection. In physically demanding occupations, the reduction in pillar pain and grip strength limitation appeared particularly relevant. Several cohorts documented earlier progression back to manual duties than would be expected after open or endoscopic release [64]. Although RTW definitions varied between studies, most reports described earlier functional use of the hand when real-time imaging and minimal access are combined. Preoperative ultrasound parameters, including nerve morphology, stiffness, and vascularity, may also influence baseline symptom burden and recovery potential, although this relationship remains incompletely defined in the current CTR outcome literature.

### 7.3. Operator Learning Curve and Adoption

US-CTR was initially met with scepticism, particularly regarding the level of sonographic expertise required. Early adopters reported that while imaging acquisition and instrument handling required structured training, the technique was learnable and reproducible with appropriate proctorship [22,65]. Subsequent reports suggested that outcomes become more predictable with experience, although operator training and case volume remained important considerations.

### 7.4. A Platform for Further Innovation

US-CTR also provided the technical basis for later ultraminimally invasive systems. Emphasis on small access points and continuous anatomical visualisation informed the development of thread-based, hook-knife and needle-mounted blade techniques. These approaches rely on the same underlying principle established by US-CTR: maintaining visualisation of critical structures allows access to be reduced without compromising procedural safety.

## 8. Ultraminimally Invasive Techniques

As ultrasound guidance became more widely adopted in CTR, attention shifted to how much access was required to achieve complete ligament release. Several groups investigated whether decompression could be performed through puncture-sized entry points while maintaining continuous visualisation of the median nerve and TCL. These approaches, later described as ultraminimally invasive (Ultra-MIS), were developed primarily to limit soft-tissue disruption rather than to modify established open or endoscopic workflows. These ultraminimal approaches are inseparable from advances in ultrasound guidance, as continuous imaging is required to safely execute ligament division through puncture-sized access while confirming anatomical relationships in real time. In contrast to endoscopic techniques, Ultra-MIS techniques do not rely on optical portals or rigid scopes. Instead, they use fine cutting elements, such as looped threads or small hook-type blades, introduced percutaneously under real-time ultrasound guidance. Early anatomical and clinical reports shared a common objective: to divide the TCL while preserving adjacent structures and minimising surface trauma [21,22].

### 8.1. Thread-Based Percutaneous Release

Guo and colleagues [67] described one of the first reproducible thread-based percutaneous techniques, demonstrating TCL division using looped surgical thread passed through a small percutaneous route under ultrasound guidance. The method relied on controlled friction rather than a cutting blade, with continuous imaging used to confirm the thread position relative to the median nerve. Cadaveric validation showed complete ligament transection without observed neurovascular injury, and subsequent clinical series reported symptom improvement following the procedure [67,68]. Further anatomical and clinical evaluation by Burnham and colleagues confirmed that the thread could be positioned reproducibly using established sonographic landmarks [69,70,71]. In these reports, functional recovery was assessed within treated cohorts; it was seen that RTW durations were reduced, demonstrating that incisionless decompression is feasible in clinical practice.

### 8.2. Hook-Knife and Micro-Blade Approaches

In parallel, Rojo-Manaute and co-workers introduced an ultrasound-guided hook-knife technique that enabled TCL division through a puncture-sized access point [22,23]. Cadaveric and pilot clinical studies confirmed that complete release could be achieved under continuous ultrasound visualisation. The authors later applied the term ultraminimally invasive to distinguish these techniques from mini-open and endoscopic approaches with emphasis placed on access size rather than conceptual novelty. In doing so, they effectively coined the term Ultra-MIS [21]. Vanni et al. described a related dual-tunnels micro-access technique designed to optimise visual control during decompression [14]. Their clinical series reported full symptom relief and a mean RTW interval of seven to ten days [14], confirming the practicality of same-day outpatient decompression under local anaesthesia. These methods aimed to achieve TCL division with only percutaneous access [72].

### 8.3. Functional Recovery and Return-to-Work

Across published Ultra-MIS reports, postoperative recovery was reported using a combination of symptom resolution, clinical examination and cohort-specific RTW intervals. In these studies, RTW durations commonly fell within 7 to 14 days, although definitions varied and occupational factors remained influential [14,21,23,68,71,72]. Across studies, the consistent signal was not the exact RTW duration but the direction of effect: reducing access size and limiting palmar dissection reliably translated to earlier functional use of the hand when compared to traditional open approaches. Occupational demands and workplace constraints continued to influence the timing of RTW. Even so, the procedural contribution to recovery was clear and reproducible. This is highlighted by reported patient satisfaction above 90% and improvements in both functional status and symptom severity.

### 8.4. Position Within the Carpal Tunnel Release Development Pathway

Ultra-MIS techniques occupy a transitional position between ultrasound-guided limited-access procedures and fully microinvasive systems. They preserve continuous visualisation while reducing access to the minimum required to manipulate the cutting element safely. These approaches demonstrated that completeness of decompression could be confirmed without wide exposure, while also revealing practical constraints related to the manual control of externally applied cutting instruments [19,20,73]. Addressing these constraints informed the subsequent development of self-contained needle-mounted systems, discussed in the following section.

## 9. Microinvasive and Needle-Mounted Blade Techniques

The most recent phase of CTR has focused on further reducing the access size while retaining full control over ligament division. Microinvasive CTR (Micro-CTR) techniques were developed in response to the practical limitations of thread-based and hook-knife systems, particularly the reliance on external tensioning or extended manual blade manipulation. The defining feature of Micro-CTR is the integration of needle-mounted, retractable cutting elements with continuous ultrasound guidance, allowing controlled ligament division through a single puncture while maintaining uninterrupted visualisation of critical anatomy.

### 9.1. Development of Needle-Mounted Blade Systems

Hebbard first described an ultrasound-guided Micro-CTR system in the late 2010s, using a retractable needle-mounted blade housed within an 18-gauge needle cannula [19]. In a cadaveric model, the authors demonstrated complete TCL division through a sub-2 mm puncture while maintaining continuous visualisation of the median nerve and adjacent structures. The blade was deployed only during active cutting and remained shielded within the needle cannula during advancement and withdrawal, directly addressing the safety concerns associated with exposed cutting elements. Subsequent clinical validation confirmed that the same system could be applied reproducibly in patients under local anaesthesia [20]. Procedures were performed in an outpatient setting, without sutures, and achieved complete decompression with minimal postoperative discomfort and negligible complication rates. Importantly, the technique preserved one of the core principles established by ultrasound-guided CTR: uninterrupted, real-time visual control throughout the release.

### 9.2. Clinical Outcomes and Return-to-Work

Clinical series evaluating Micro-CTR report some of the shortest RTW durations described for any CTR modality. Hebbard and his team [20] documented median RTW intervals of approximately one week, accompanied by minimal postoperative pain and rapid restoration of hand use. These outcomes are consistent with limited access trauma and avoidance of palmar dissection inherent to the technique. Comparative data further supported these findings. Ulusoy and colleagues compared ultrasound-guided Micro-CTR with mini-open surgery and reported faster functional recovery and earlier RTW in the microinvasive group, while maintaining equivalent symptom relief and durability at follow-up examinations [25]. In that study, Micro-CTR achieved RTW durations in the five-to-ten-day range, compared with longer intervals following mini-open and endoscopic approaches.

### 9.3. Procedural Efficiency and System-Level Implications

Beyond recovery metrics, Micro-CTR alters how CTR can be delivered within healthcare systems [30,32]. The procedure can be performed under local anaesthesia, outside the operating theatre, and without endoscopic equipment. This simplifies workflow while preserving anatomical precision. Ferreira-Silva and colleagues demonstrated that ultrasound-guided thread- and needle-based systems could be incorporated into standardised clinical pathways, supporting the feasibility of Micro-CTR as a routine intervention rather than a niche technique [73]. Reduced anaesthetic exposure, shorter procedure times, and earlier RTW collectively contributed to reduced indirect costs, particularly in working-age populations. These characteristics align Micro-CTR with the value-based surgical principles discussed earlier.

### 9.4. Scope Beyond Carpal Tunnel Release

The conceptual strength of needle-mounted blade systems lies in their applicability across anatomical contexts. Vega and colleagues applied a blade-tipped needle system to ultrasound-guided microinvasive trigger-thumb release, demonstrating the feasibility of this approach in other focal entrapment conditions ([74]: Although anatomically focused on trigger-thumb release, the study by Vega et al. [74] was included because the technology is relevant, demonstrating the same needle-mounted, blade-tipped system that underpins emerging microinvasive Carpal Tunnel Release designs). While anatomically distinct from CTR, the study results reinforced the broader utility of controlled, image-guided ligament division through puncture sized access.

### 9.5. Position of Microinvasive Carpal Tunnel Release in Contemporary Practice

Micro-CTR represents the smallest access, image-guided decompression technique with published clinical validation to date. It consolidates several decades of CTR development into a single operative principle, fundamentally realising the principle first proposed by Learmonth [36]: complete median nerve decompression achieved with minimal tissue disruption and continuous visual oversight. Unlike earlier minimally invasive approaches, Micro-CTR does not depend on external cutting tension or extended instrument trajectories. Decompression is delivered through a self-contained system designed for precision and reproducibility. At present, Micro-CTR defines the minimum practical threshold of invasiveness in CTR. Further reductions in access size are unlikely to yield proportional gains in recovery without addressing non-procedural determinants of RTW. In this context, Micro-CTR serves as a logical endpoint for surgical refinement and provides a stable reference point for the comparative synthesis that follows.

## 10. Comparative Synthesis: Return-to-Work as a Core Functional Metric

RTW provides an integrative outcome for comparing CTR techniques because it reflects not only symptom resolution, but also functional recovery, pain tolerance, occupational demand, and the practical consequences of surgical design. Unlike isolated clinical scores, RTW captures the point at which decompression is expressed as usable hand function within real-world constraints. Across the evolution of CTR, RTW has progressively shifted from a secondary observation to a central comparative metric.

### 10.1. Evolution of Return-to-Work Across Carpal Tunnel Release Modalities

Early OCTRs established a benchmark for surgical success based on durable symptom relief but recovery was prolonged. Large historical series reported RTW intervals commonly extending six to eight weeks, largely driven by incision morbidity, pillar pain, and delayed grip tolerance [39,40,41]. Later observational studies reported that traditional OCTR required on average four to six weeks of work absence with improved procedural technique and instrumentation [9,44]. These intervals became the reference standard against which subsequent techniques were judged.

The introduction of ECTR represented the first major inflexion point. Randomised and multicentre studies consistently demonstrated that equivalent decompression could be achieved with earlier functional recovery, reducing RTW to approximately two to four weeks [45,46,49,51,53,54]. This reduction occurred without compromising long-term outcomes, suggesting that access size and tissue disruption, not decompression completeness, were the primary contributors to recovery duration.

Ultrasound-guided CTR further compressed the recovery window by eliminating formal incisions and sutures, while preserving continuous visualisation of the TCL and adjacent neurovascular structures. Across prospective cohorts and registry studies, median RTW durations clustered between 10 and 21 days, with the majority of patients resuming work within three weeks [15,63,64,66]. These findings reinforced the association between procedural precision, minimal soft-tissue disruption, and earlier RTW.

**Figure 1:**
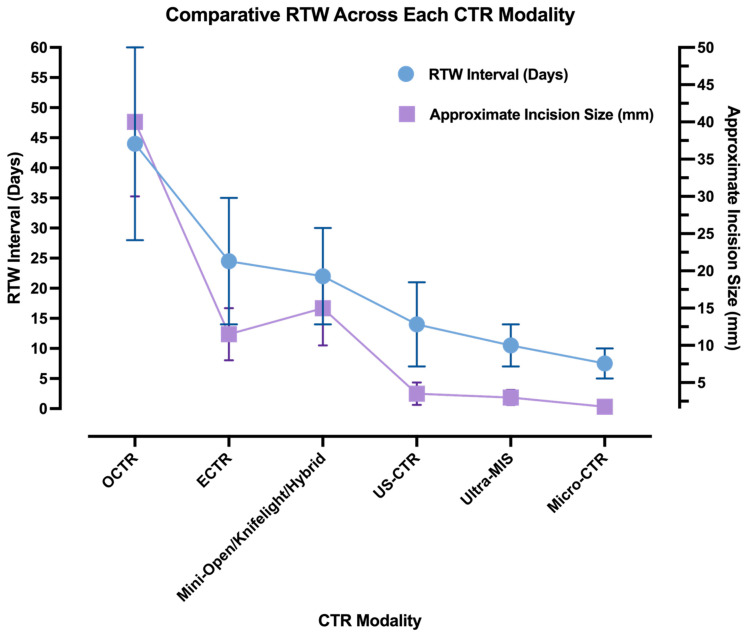
Illustrative trend in RTW duration (blue) and incision size (lavender) across the historical progression of CTR modalities. Data points reflect minimum and maximum values from the representative literature.

The emergence of Ultra-MIS and Micro-CTR has shifted expectations again. Thread-based, hook-knife, and needle-mounted blade systems consistently reported RTW intervals measured in days rather than weeks, with many patients returning to light or full duties within one to two weeks [20,21,23,25,68,71]. Figure 1 summarises the reduction in median RTW from a duration of several weeks to a matter of days. At this stage, further reductions in RTW appear to be driven by access minimisation and anaesthetic strategy rather than incremental refinements in decompression technique (Table 1).

### 10.2. Return-to-Work as a Functional and Socioeconomic Indicator

Across modalities, RTW shortened in parallel with decreasing invasiveness, forming a clear gradient from open surgery to microinvasive release. This pattern is reproducible across study designs and healthcare systems, suggesting that the relationship is not incidental. Reduced incision size, avoidance of palmar dissection, and local anaesthesia consistently reduced postoperative pain, accelerated functional use of the hand, and reduced reliance on prolonged rehabilitation.

**Figure 2:**
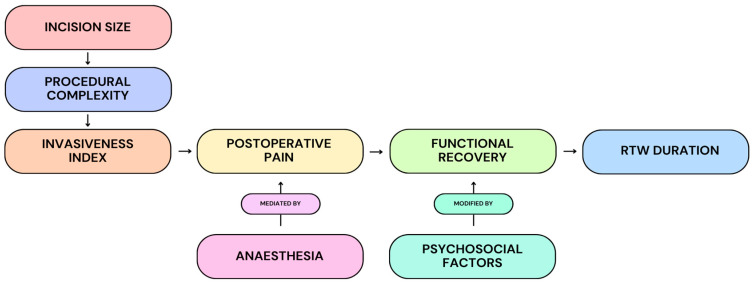
Conceptual model illustrating the proposed relationship between incision size, procedural complexity, and representative invasiveness to postoperative pain, functional recovery, and ultimately RTW duration. Anaesthesia is depicted as a mediator of postoperative pain, while psychosocial factors are shown as modifiers of recovery trajectories. This framework contextualises how procedural design may influence functional and socioeconomic outcomes within the observational literature.

At the same time, RTW is not a purely surgical endpoint. Multiple studies demonstrated that occupational demands, psychosocial readiness and workplace flexibility remained influential modifiers of recovery timelines (Figure 2) [3,5,11,12,34,35,75]. Manual labour, rigid duty requirements, and compensation frameworks can delay RTW even when medical recovery is complete. However, these factors modify rather than negate the procedural effect: less invasive techniques reliably shorten the earliest feasible return to function. From a systems perspective, RTW also functions as a surrogate for indirect economic costs. Shorter work absence reduces wage loss, compensation expenditure, and downstream healthcare utilisation [30,32]. As CTR increasingly shifts toward outpatient and office-based delivery, RTW becomes a practical marker of cost-effective care, linking procedural efficiency to societal benefit.

### 10.3. Implications for Comparative Evaluation

Taken together, the comparative literature supports RTW as a meaningful benchmark for evaluating CTR techniques across eras. While symptom scores and nerve-conduction studies confirm decompression success, RTW captures how effectively that success is reflected in functional recovery and work participation. The progressive shortening of RTW from weeks to days mirrors the broader surgical transition from exposure-based approaches to image-guided precision. Microinvasive and needle-mounted blade systems currently define the minimum bound of reported recovery time, without evidence of compromised safety or durability. Whether further reductions in RTW are achievable through additional technical refinement remains uncertain. The largest gains to date appear to have resulted from access minimisation, visualisation, and anaesthetic strategy rather than further reductions in size.

## 11. Discussion

### 11.1. Reframing Success Through Return-to-Work

The primary contribution of this review was the cross-study synthesis of RTW as a functional benchmark across CTR modalities, highlighting how access size and visualisation strategy collectively shape the earliest functional recovery. Across modern CTR techniques, improvements in RTW closely parallel advances in imaging-based visualisation, underscoring the role of ultrasound not merely as an adjunct, but as a driver of procedural refinement and recovery acceleration. Through the history of CTR, surgical success was traditionally defined by symptom resolution and anatomical decompression. While these outcomes remain necessary, they are no longer sufficient. The comparative literature reviewed here demonstrates that RTW represents the endpoint at which decompression is associated with functional hand use under real-world constraints. Unlike symptom scores or nerve-conduction studies, RTW reflects recovery as it is experienced by patients, employers, and healthcare systems alike. The progressive reductions in RTW from four to six weeks after open release to under two weeks with Ultra-MIS and minimally invasive techniques are unlikely to be incidental. It appears to mirror the stepwise reduction in tissue disruption through smaller access routes, improved visualisation, and local anaesthetic strategies [15,20,39,40,45]. In this context, RTW functions as a composite outcome, integrating pain resolution, dexterity, confidence in hand use, and occupational readiness into a single, observational milestone.

### 11.2. Technical Refinement as the Primary Driver of Recovery Acceleration

The evolution of CTR techniques demonstrates a consistent principle: when decompression quality is preserved, recovery time is largely determined by how the ligament is accessed rather than whether it is fully released. Open techniques prioritised exposure at the expense of palmar tissue trauma, while endoscopic systems demonstrated that equivalent decompression could be achieved with reduced dissection and earlier functional recovery [13,14,45,49,53]. Ultrasound-guided CTR advanced this concept further by replacing optical access with dynamic imaging, thereby reducing the need for formal incisions [15,18,21,22].

Microinvasive needle-mounted blade systems represented the logical extension of this trajectory. By confining cutting to the ligament itself and shielding surrounding structures within a cannula, these devices minimise collateral tissue injury while maintaining continuous visual control [19,20]. RTW intervals of five to ten days are not anomalous [24,25,76]; they are consistent with the reduction in surgical trauma to its minimum practical threshold.

### 11.3. The Limits of Further Miniaturisation

While the trend toward smaller access has been associated with substantial gains in recovery speed, the literature suggests that further reductions in RTW may not scale proportionally with additional miniaturisation. Once incisions are replaced with puncture-sized access and procedures are performed under local anaesthesia, the remaining delays in RTW are increasingly shaped by non-surgical factors. Surgery alone cannot resolve this. Occupational demands, compensation frameworks, and psychosocial readiness consistently modify timelines across all CTR modalities [5,12,35]. This observation reframes the next phase of CTR optimisation. Rather than pursuing even smaller access points, future gains in RTW are more likely to come from aligning surgical technique with preoperative counselling, workplace accommodation, and realistic recovery expectations. In this sense, much of the procedure-related acceleration in recovery has likely already been achieved.

### 11.4. Return-to-Work as a Value-Based Outcome Measure

RTW also provides a direct link between clinical outcomes and health-economic value. Shorter work absence is associated with reduced indirect costs related to wage loss, compensation claims, and prolonged healthcare utilisation [30,32]. As CTR increasingly shifts toward office-based and ambulatory settings, techniques that reliably shorten RTW align closely with value-based care models. Importantly, this does not imply that the fastest technique is universally appropriate. Open and endoscopic CTR remain effective and durable options, particularly in settings where imaging expertise or equipment is limited. However, when RTW is prioritised as a core outcome, minimally invasive and microinvasive approaches demonstrated a clear functional advantage in the published cohorts, without sacrificing safety or efficacy.

### 11.5. Implications for Clinical Practice and Research

The findings of this review support the reframing of CTR evolution. Rather than asking whether a technique works, the more relevant question is how efficiently it restores function and occupational participation. RTW provides a practical answer to that question. Its consistent shortening across successive CTR innovations reflects the importance of access minimisation, visualisation, and anaesthetic strategy in shaping recovery. Future research should focus less on proving equivalence in symptom relief, which is already well established, and more on standardising RTW definitions and stratifying outcomes by occupational demand. Clear distinctions between light-duty and full-duty RTW would improve comparability across studies and strengthen meta-analytic synthesis. Integrating procedural variables with psychosocial and workplace factors may also enable more accurate prediction of individual recovery trajectories.

This review has limitations inherent to the available literature. RTW is variably defined (e.g., light-duty versus full-duty), occupational stratification is inconsistent, and many cohorts are influenced by compensation and workplace policy, which limits comparability across modalities. These constraints support the need for standardised RTW reporting frameworks in future CTR studies.

## 12. Conclusion and Future Directions

### 12.1. Summary of Evidence

Over more than a century of surgical refinement, CTR has progressed from wide open exposure to image-guided microinvasive decompression. This evolution has not altered the fundamental therapeutic objective, complete division of the TCL, but it has profoundly changed how recovery unfolds. The evidence reviewed here consistently reports a reduction in RTW duration as access size has narrowed, visualisation has improved, and anaesthetic strategies have shifted toward local, outpatient delivery [15,20,39,40,45]. Across open, endoscopic, ultrasound-guided, ultraminimally invasive, and microinvasive techniques, RTW has shortened from weeks to days without clear evidence of compromised symptom relief, safety or durability. Taken together, these findings suggest that, when decompression quality is preserved, recovery durations align more with access-related tissue disruption and perioperative strategy than with decompression itself.

### 12.2. Return-to-Work as a Definitive Functional Endpoint

RTW has emerged as one of the more informative functional endpoints in CTR because it integrates pain resolution, hand function, confidence in use, and occupational readiness into a single, observable outcome. Unlike isolated clinical metrics, RTW provides a pragmatic benchmark for comparing techniques across healthcare systems and work environments. Importantly, multiple studies indicate that RTW is not a purely technical outcome. Occupational demands, compensation status, and psychosocial factors continue to influence when patients return to work, even when medical recovery is complete [12,35]. These influences do not diminish the value of RTW; rather, they reinforce its relevance as a composite endpoint that reflects both biological recovery and real-world constraint.

### 12.3. Clinical Implications

From a clinical perspective, the findings of this review support a shift in how CTR success is discussed with patients and stakeholders. While open and endoscopic CTR remain effective and durable, image-guided minimally invasive and microinvasive techniques have been associated with faster recovery speeds and early return to function. For working-age populations, particularly those for whom time away from work carries substantial economic or personal cost, RTW should be considered alongside traditional clinical outcomes.

Microinvasive needle-mounted blade systems currently represent the shortest reported recovery time to date, achieving complete decompression through puncture-sized access under real-time imaging with RTW commonly measured in days rather than weeks [20,25]. These findings suggest that further gains in recovery are achievable without sacrificing procedural control or safety in appropriately selected settings.

### 12.4. Future Directions: Recovery Pathways and Return-to-Work

The next phase of CTR advancement is unlikely to be defined by further reductions in access size alone. Instead, progress will likely depend on how effectively surgical innovation is integrated with perioperative care, patient counselling, and workplace accommodation. Standardising RTW definitions, particularly distinguishing between light-duty and full-duty return, would improve comparability across studies and facilitate the translation of research findings into clinical guidance. Future studies may benefit from prioritising multicentre data collection that combines procedural detail with occupational and psychosocial variables. Such integration could enable more accurate prediction of recovery trajectories and allow RTW to function not only as a retrospective outcome but as a tool for expectation management and shared decision-making.

### 12.5. Closing Statement

The transition from OCTR to image-guided Micro-CTR reflects a broader shift in surgical thinking, from exposure toward precision, and from symptom resolution toward functional recovery. By placing RTW at the centre of comparative evaluation, this review highlights how technical refinement has been associated with meaningful gains for patients, employers, and healthcare systems. In contemporary CTR practice, success is increasingly defined not only by decompression completeness, but also by how efficiently and reliably patients return to work and resume their lives.

## Figures and Tables

**Table 1:** Comparative RTW intervals reported across major CTR modalities. Data derived from multicentre and randomised studies demonstrating reduction in median RTW durations in cohorts undergoing less invasive procedures.

CTR Modality	Representative Studies	Typical RTW Interval	Key Determinants of RTW	Clinical/Economic Implications
Open Carpal Tunnel Release (OCTR)	[9,40,41,44]	4–6 weeks (28–42 days)	Wound healing, pillar pain, occupation type	Baseline for efficacy; prolonged convalescence and work loss.
Endoscopic CTR (ECTR)	[45,46,49,53,54]	2–4 weeks (14–28 days)	Technique mastery, pillar pain, nerve irritation	Faster RTW and reduced morbidity; equivalent long-term outcomes to OCTR.
Mini-Open/KnifeLight/Hybrid	[56,57,58]	2–3 weeks (14–21 days)	Incision length, scar tenderness	Transitional approach toward minimally invasive CTR.
Ultrasound-Guided CTR (US-CTR)	[15,24,63,64,66]	10–21 days	Sonographic precision, local anaesthesia, job autonomy	Outpatient feasibility; >90% RTW within 3 weeks; reduced indirect cost.
Ultraminimally Invasive CTR (Ultra-MIS)	[14,23,67,68,71,72]	7–14 days	Micro-access safety, thread tension control	Halves convalescence time compared to ECTR; minimal scar and pain.
Microinvasive CTR (Micro-CTR)	[19,20,25,73]	5–10 days	Needle trajectory control, ultrasound guidance, operator training	Fastest RTW documented; outpatient local anaesthesia; optimised cost-effectiveness.

## Data Availability

Data sharing is not applicable to this article as no datasets were generated or analysed during the current study. All data supporting this review are contained within the article and its reference list.

## Abbreviations

The following abbreviations are used in this manuscript:

CTS	Carpal Tunnel Syndrome
CTR	Carpal Tunnel Release
OCTR	Open Carpal Tunnel Release
ECTR	Endoscopic Carpal Tunnel Release
US-CTR	Ultrasound-Guided Carpal Tunnel Release
Ultra-MIS	Ultraminimally Invasive Surgery
Micro-CTR	Microinvasive Carpal Tunnel Release
RTW	Return-to-Work
TCL	Transverse Carpal Ligament
